# Adenosine Receptors and Wound Healing, Revised

**DOI:** 10.1100/tsw.2006.194

**Published:** 2006-08-17

**Authors:** Bruce M. Cronstein

**Affiliations:** Department of Medicine, Pathology and Pharmacology, New York University School of Medicine, New York, NY 10016, USA

**Keywords:** Adenosine, adenosine receptor, angiogenesis, inflammation, granulation tissue

## Abstract

Recent studies have demonstrated that application of topical adenosine A2A receptor agonists promotes more rapid wound closure and clinical studies are currently underway to determine the utility of topical A_2A_ adenosine receptor agonists in the therapy of diabetic foot ulcers. The effects of adenosine A_2A_ receptors on the cells and tissues of healing wounds have only recently been explored. Here we summarize the evidence indicating that adenosine and selective adenosine agonists, acting at A_2A_ receptors, promote the salutary functions of inflammatory cells, endothelial cells and fibroblasts in stimulating wound healing.

## INTRODUCTION

Wound repair is an essential homeostatic mechanism that involves a series of coordinated and overlapping phases: inflammation, angiogenesis, new tissue generation, and tissue reorganization. Aberrant or inadequate wound repair contributes to the disability suffered by patients with diabetes mellitus and is a major problem for patients suffering from venous insufficiency. Our laboratory has recently discovered that topical application of adenosine $A_{2A}$ receptor agonists promotes more rapid wound healing in full-thickness dermal wounds in both normal animals and animals with streptozotocin-induced diabetes mellitus[1]. More recently, Investigational New Drug status has been granted to the highly selective adenosine $A_{2A}$ receptor agonist MRE0094 for use in the treatment of poorly healing foot ulcers in diabetics and initial dose-ranging trials of the drug in patients have begun. To better translate this finding to the clinic, we will review the mechanisms by which adenosine receptor agonists affect the cells involved in wound healing and, thus, promote wound healing.

## PHYSIOLOGIC AND PHARMACOLOGIC ROLES OF ADENOSINE

Adenosine was first recognized as a physiologic regulator of coronary vascular tone by Drury and Szent-Gyorgy in 1929[2], however it was not until 1970 that Sattin and Rall showed that adenosine regulates cell function via occupancy of specific receptors on the cell surface[3]. It is now clear that there are at least four different subtypes of adenosine receptor, any one or combination of which may be expressed on the cell surface (recently reviewed in [4]). Four adenosine receptors have been cloned and the deduced sequence reveals that all four are members of the large family of 7-transmembrane spanning, G protein coupled receptors. Three of the adenosine receptor subtypes — $A_1$, $A_{2A}$, and $A_{2B}$ — are highly conserved throughout evolution (80–95% sequence homology) whereas $A_3$ receptors vary significantly among species. In general, $A_1$ receptors are coupled to pertussis toxin-inhibited $G_i$ coupled signal transduction proteins or directly to ion channels whereas $A_2$ receptors ($A_{2A}$ and $A_{2B}$) are coupled to $G\alpha_S$-linked receptors and stimulate adenylyl cyclase and cAMP accumulation. Adenosine receptors or receptor-mediated effects have been demonstrated in virtually every tissue or organ examined. Some of the more prominent physiologic or pharmacologic effects mediated by adenosine receptors include: neurotransmission ($A_1$ and $A_{2A}$ receptors, the CNS effects of caffeine are thought to result from adenosine receptor antagonism); modulation of cardiac conduction ($A_1$ receptors, intravenous preparations of adenosine are licensed for clinical use for the treatment of supraventricular tachycardia); coronary vasodilation ($A_{2A}$ receptors, infusions of adenosine are licensed for clinical use as a coronary vasodilator for pharmacologic stress testing); regulation, indirectly, of airway tone ($A_{2B}$ receptors); inhibition of inflammation (mediates the anti-inflammatory effects of low-dose methotrexate, the most commonly used second-line agent for the treatment of rheumatoid arthritis (reviewed in [5,6]). Our laboratory first demonstrated that adenosine modulates neutrophil function via interaction with $A_1$ and $A_2$ receptors and, when released from cells in increased concentration, inhibits inflammation (reviewed in [6]). In many tissues and cell types, $A_1$ and $A_{2A}$ receptors have opposing actions not only on cAMP levels but on function as well (cf. [7,8]). More recently, as noted above, topical application of an adenosine $A_{2A}$ receptor agonist has been shown to promote wound healing, a process dependent upon the proliferation of blood vessels, in both normal mice and diabetic rats[1].

## THE EFFECTS OF ADENOSINE $A_{2A}$ RECEPTOR AGONISTS ON WOUND HEALING

Our lab has recently reported that topical application of an adenosine $A_{2A}$ receptor agonist increases the rate at which wounds close[1]. That adenosine $A_{2A}$ receptors were involved in this pharmacologic effect was demonstrated by the observation that a specific adenosine $A_{2A}$ receptor antagonist, but not antagonists at other adenosine receptors, reversed the effect of the selective $A_{2A}$ receptor agonist CGS21680 in these studies. Treatment of wounds with an adenosine receptor agonist promoted fibroblast migration *in vitro* and in the adenosine receptor agonist-treated mice there was an increase in matrix and fibroblast infiltration into the wounds[1]. More recent studies demonstrate that a more highly selective adenosine $A_{2A}$ receptor agonist, MRE0094, is a more potent promoter of wound healing than recombinant platelet derived growth factor (becaplermin[9]). The role of adenosine $A_{2A}$ receptors in the promotion of wound healing was more fully confirmed by the observation that a selective adenosine $A_{2A}$ receptor agonist promotes wound healing in wild type but not $A_{2A}$ receptor knockout mice[6]. In these studies, there was a marked increase in the number of blood vessels in the healing wounds of wild type mice treated with the adenosine $A_{2A}$ receptor agonist as compared to untreated controls. Absence of $A_{2A}$ receptors was associated with disorganized granulation tissue although wound closure was not delayed in the knockout mice. In contrast to the work showing that the adenosine $A_{2A}$ receptor promotes wound healing, Sun and colleagues observed that $N^6$cyclopentyladenosine, a relatively selective $A_1$ receptor agonist, promotes wound healing and, interestingly, hair growth[10]. In this study, there was no confirmation that the high concentrations of the agonist used were indeed selective for $A_1$ receptors or whether the phenomenon was mediated by adenosine acting at $A_{2A}$ receptors. Interestingly, more recent studies have suggested that the capacity of minoxidil to stimulate hair growth is mediated by ligation of adenosine $A_1$ receptors by increased concentrations of endogenously released adenosine[11]. The literature reviewed here indicates that adenosine, acting at $A_{2A}$ receptors, stimulates the functions of inflammatory cells, endothelial cells and fibroblasts that promote wound healing.

## THE EFFECTS OF ADENOSINE ON INFLAMMATION

As noted above, inflammation is the first step in wound healing. During this phase of tissue repair, necrotic debris is eliminated and bacterial superinfection is prevented by an influx of inflammatory cells, primarily neutrophils at the outset with a predominance of monocytes and lymphocytes later during the course of wound healing. In addition to clearing debris, inflammatory cells release a variety of agents that stimulate the later stages in wound healing. In some pathologic settings, persistent or overly exuberant inflammation may interfere with wound healing. Our laboratory first demonstrated that adenosine, acting at a specific extracellular receptor, inhibits stimulated neutrophil function, release of reactive oxygen species[12,13]. In subsequent studies, it was demonstrated that adenosine receptor occupancy inhibits neutrophil phagocytosis, stimulated neutrophil adhesion to endothelial cells and matrix proteins, and neutrophil-mediated endothelial cell injury[7,14,15]. Early studies demonstrated, by pharmacologic means, that neutrophils express at least two different types of adenosine receptor, one of which ($A_2$ receptors) mediates the inhibition of neutrophil function described above and the other ($A_1$ receptors), when occupied, promotes neutrophil chemotaxis, phagocytosis, and adhesion to vascular endothelial cells[7,14,15,16,17]. These observations have now been confirmed and expanded upon by a large number of laboratories (reviewed in [18]) and it is clear that $A_{2A}$ receptors are the $A_2$ receptors responsible for the suppression of neutrophil function. Although the actions of adenosine $A_{2A}$ receptor occupancy on neutrophil function have generally been ascribed to cAMP (cf. [19]) this signal transduction mechanism appears to hold only in primed (tumor necrosis factor- or endotoxin-stimulated) neutrophils. In contrast, in unprimed neutrophils, the effects of adenosine $A_2$ receptor occupancy modulates neutrophil behavior by a cAMP-protein kinase A-independent mechanism involving activation of a plasma membrane-associated protein phosphatase[20,21].

In addition to its effects on neutrophil function, adenosine — acting at its receptors — also modulates macrophage function. In contrast to neutrophils, both $A_{2A}$ and $A_3$ adenosine receptors appear to be involved in suppression of inflammatory functions of macrophages and there are species-related differences in the effects of these adenosine receptors on macrophage function. Adenosine, acting at $A_{2A}$ receptors, inhibits generation of reactive oxygen species, phagocytosis, cytokine release (TNF, C2, tissue factor/procoagulant), and increases plasminogen activator and IL-10 production (reviewed in [18]). Similar effects have been described for the $A_3$ receptor, although the $A_3$ receptor contribution to adenosine-mediated modulation of inflammation is most notable in cells from mice and is often not demonstrable in human cells.

Adenosine is released from cells and tissues following a variety of insults, e.g., ischemia or inflammation, and presumably is derived from the dephosphorylation of ATP either within the cell or extracellularly[22,23,24,25]. Previous studies, using relatively nonselective adenosine receptor antagonists, have shown that endogenous adenosine regulates inflammation[26] and more recent studies have demonstrated that absence of adenosine $A_{2A}$ receptors increases inflammatory injury[27].

Clearly the use of adenosine or adenosine analogues for the treatment of inflammation has been contemplated and adenosine $A_{2A}$ receptor agonists have been shown to be useful in several models of inflammation[28,29,30,31,32,33]. Nonetheless, fear of the many potential physiologic and pharmacologic effects of adenosine receptor agonists has limited the utility of this approach to suppressing inflammation. Another approach to ligation of adenosine receptors as an anti-inflammatory agent is to promote endogenous adenosine release. The first demonstration that existing anti-inflammatory therapies might make use of endogenous adenosine was reported in 1991 when it was shown, based on *in vitro* experiments, that low-dose methotrexate therapy, the most commonly used and effective second-line therapy for the treatment of rheumatoid arthritis, induces an increase in adenosine release from injured cells and the adenosine so released inhibits inflammation[34]. Subsequent studies provided support for the hypothesis that adenosine mediates the anti-inflammatory effects of methotrexate provided by observations in an *in vivo* model of inflammation in which adenosine $A_2$ receptor antagonists and adenosine deaminase reversed the anti-inflammatory effects of methotrexate in mice[35]. In further animal studies, it has been observed that a commonly used adenosine receptor antagonist, caffeine, reverses the anti-inflammatory effects of methotrexate in the adjuvant arthritis model of inflammation[36] and coffee ingestion (presumably with caffeine) was recently shown to interfere with the anti-inflammatory actions of methotrexate in patients as well[37], an observation that suggests that avoidance of caffeine may enhance the therapeutic effects of methotrexate. In other studies, sulfasalazine, another commonly used second-line agent for the therapy of inflammatory arthritis, shares a mechanism of action with methotrexate; sulfasalazine also promotes the release of adenosine from inflamed cells and tissues and the adenosine released occupies $A_2$ receptors on inflammatory cells to diminish inflammation[38]. On the basis of these findings and confirmatory studies from other laboratories, specific enzyme (adenosine

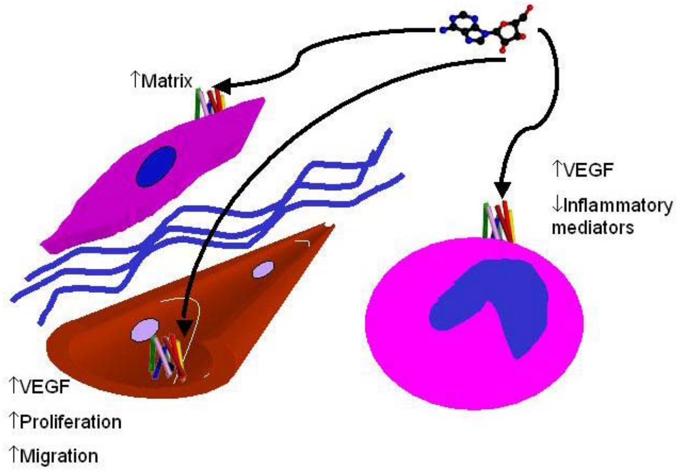

**FIGURE.** The effect of adenosine, acting at $A_{2A}$ receptors, on the cells involved in wound healing. VEGF: vascular endothelial cell growth factor.

kinase) inhibitors have been developed that promote adenosine release and suppress inflammation[39,40,41,42].

Interestingly, FK506, a potent and commonly used immunosuppressive agent, has also been reported to inhibit adenosine kinase and adenosine is thought to mediate its effects on acute inflammation[43]. None of the agents described above have been studied in the promotion of wound healing, nor have there been reports that administration of either sulfasalazine or methotrexate interferes or promotes wound healing.

## THE EFFECT OF ADENOSINE RECEPTOR OCCUPANCY ON ANGIOGENESIS AND ENDOTHELIAL CELL FUNCTION

Previous reports document, by RT-PCR, that cultured human endothelial cells (umbilical vein) express message for all four adenosine receptors[1], although more recent studies document only $A_{2A}$ and $A_{2B}$ receptor message in human microvascular endothelial cells (Khoa, in press). Although the functional effects of ligation of all of the adenosine receptors on endothelial cells have not been fully explored, it is now clear that adenosine receptor occupancy promotes several potential angiogenic effects when studied *in vitro*. These studies, from different laboratories, demonstrate that adenosine, acting at specific $A_2$ receptors, promotes endothelial cell proliferation, migration, and growth factor (VEGF) production[44,45,46,47,48,49]. Moreover, in a recent study it was shown that the effect of adenosine on endothelial cell proliferation is mediated by $A_{2A}$ receptors and that cAMP is probably not the second messenger for this phenomenon[49]. Bouma and co-workers have also reported that adenosine, possibly acting at $A_3$ receptors, diminishes expression of IL-8, a potent angiogenic agent, by stimulated endothelial cells[50]. In contrast, others have reported that the cells responsible for the most abundant IL-8 secretion at inflamed sites, macrophage/monocytes, are unaffected by adenosine receptor agonists in their capacity to secrete this angiogenic cytokine[51,52]. Adenosine, acting at $A_{2A}$ and $A_3$ receptors, inhibits tissue factor expression on endothelial cells[53] and promotes NO release by endothelial cells[54]. Recent studies suggest that adenosine $A_{2B}$ receptor occupancy is responsible for promotion of retinal angiogenesis[55,56] although these results may not apply to other vascular beds or to angiogenesis in wounds. Thus, it was recently reported[57] that mice with genetically disrupted $A_{2A}$ receptors (knockouts) form significantly fewer microvessels in healing wounds and in response to mechanical trauma (formation of an air pouch[57]). Furthermore, application of an $A_{2A}$ receptor agonist to wounds increases microvessel formation as compared to vehicle-treated mice, observations that provide the first *in vivo* evidence that adenosine $A_{2A}$ receptor occupancy promotes angiogenesis. Further studies indicate that the angiogenic effects of adenosine $A_{2A}$ receptor occupancy are mediated both directly on endothelial cells (increased endothelial cell migration and microvascular endothelial cell VEGF production) ([1, 58] and indirectly via promotion of VEGF production by macrophages[59]. Desai and colleagues [60] have also reported evidence to indicate that adenosine $A_{2A}$ receptor occupancy suppresses production of thrombospondin I, a potent inhibitor of angiogenesis and this inhibition is responsible for enhanced vascular tube formation *in vitro*. Similar to other angiogenic agents, adenosine — acting at $A_{2A}$ receptors — is a potent vasodilator that is also thought to mediate reactive hyperemia in skeletal muscle and the heart. Thus, adenosine, acting via $A_{2A}$ receptors, promotes angiogenesis both directly and indirectly and does so, notably, both *in vitro* and *in vivo*.

## THE EFFECT OF ADENOSINE $A_{2A}$ RECEPTOR ACTIVATION ON MATRIX PRODUCTION BY FIBROBLASTS

Replacement of the collagenous matrix of the skin and other tissues is an integral part of wound healing. Once the debris and destroyed matrix at the site of injury are eliminated fibroblasts lay down a new matrix. This matrix may be remodeled over a longer period of time and the wound develops the characteristic appearance of a scar. Adenosine $A_{2A}$ receptor occupancy stimulates fibroblasts to synthesize types I and III collagen at an increased level similar to that induced by the growth factor TGF-b [Chan, et al, In Press, *Arth. Rheum.*]. This observation suggests the possibility that adenosine $A_{2A}$ receptors may play a role in fibrosing conditions and scarring, an hypothesis confirmed by *in vivo* experiments. Animals lacking $A_{2A}$ receptors or treated with an adenosine $A_{2A}$ receptor antagonist were protected from developing diffuse dermal fibrosis.[Chan, et al, In Press, *Arth. Rheum.*]. The role of adenosine $A_{2A}$ receptors is unclear in tissues outside of the skin. Prior studies have demonstrated that adenosine $A_{2B}$ receptors regulate production of collagen in pulmonary and cardiac fibroblasts [61-64].

## CONCLUSION

Recent studies indicate that adenosine $A_{2A}$ receptor agonists are useful in promoting more rapid wound healing in mice. Beneficial effects of adenosine $A_{2A}$ receptor agonists on all aspects of wound healing — inflammation, angiogenesis, and tissue regeneration — have been observed and probably contribute to promotion of wound closure in both normal healthy mice and diabetic animals as well. There remains an unmet need for agents that will promote wound healing in patients with diabetes and other conditions associated with poor wound healing and it is possible that topical adenosine receptor agonists may help fill this need.

## ACKNOWLEDGMENTS

This work was supported by grants from the National Institutes of Health (AR41911, GM56268, AA13336); a grant from King Pharmaceuticals Inc.; the General Clinical Research Center (M01RR00096) and by the Kaplan Cancer Center.
